# Circulating FGF19 closely correlates with bile acid synthesis and cholestasis in patients with primary biliary cirrhosis

**DOI:** 10.1371/journal.pone.0178580

**Published:** 2017-06-01

**Authors:** Zhanyi Li, Bingliang Lin, Guoli Lin, Yuankai Wu, Yusheng Jie, Xiangyong Li, Brian Ko, Yutian Chong, Jian Luo

**Affiliations:** 1 Department of Infectious Diseases, Third Affiliated Hospital of Sun Yat-sen University, Guangzhou, Guangdong, China; 2 NGM Biopharmaceuticals, Inc., South San Francisco, California, United States of America; Laval University, CANADA

## Abstract

**Background and aim:**

Bile acid (BA) synthesis in the liver is regulated by Fibroblast Growth Factor 19 (FGF19) secreted from the ileum as an enterohepatic feedback mechanism. Although FGF19 mRNA is absent in normal liver, FGF19 gene expression was reported to increase in response to both extrahepatic and intrahepatic cholestasis. The impact of upregulated FGF19 expression on BA synthesis is unclear and the overall role of circulating FGF19 and BA synthesis under cholestatic conditions needs to be further investigated.

**Methods:**

BA synthesis was directly quantified by measuring serum concentrations of 7alpha-hydroxycholest-4-en-3-one (C4), along with serum FGF19 and other parameters, in 44 patients with primary biliary cirrhosis (PBC) and 10 healthy subjects.

**Results:**

Serum C4 were substantially lower, while those of FGF19 were higher, in cirrhotic PBC patients, as compared to those of either healthy or non-cirrhotic PBC patients. Analyses of the relationships between circulating FGF19, BA synthesis and cholestasis revealed that circulating FGF19 was strongly correlated with BA synthesis (r = -0.735, p<0.0001) and the severity of cholestasis (r = 0.590, p<0.001). Moreover, BA synthesis was found to be strongly correlated with the degree of cholestasis (r = 0.522, p = 0.0005).

**Conclusion:**

These findings demonstrate that the regulation of BA synthesis in response to cholestasis is primarily controlled by circulating FGF19 and that under cholestatic conditions, the FGF19-BA synthesis feedback mechanism remains intact. Administering FGF19, or suitable mimetic, as a pharmacological intervention to increase circulating levels of FGF19 and suppress BA synthesis by inhibiting CYP7A1 gene expression is likely to provide therapeutic benefits for many PBC patients.

## Introduction

Primary biliary cirrhosis (PBC) is a chronic disease of the liver, presumably resulting from progressive, immune-mediated destruction of the small interlobular bile ducts, leading to progressive intrahepatic cholestasis and eventually fibrosis and cirrhosis of the liver [1]. Characterized by elevated serum direct bilirubin and total bile acids (BAs), cholestasis is one of the key features of the disease [1–5], and this accumulation of hepatic BAs is believed to be central to the pathogenesis of PBC [5–7]. The excess hepatic BAs may play a substantial role in the subsequent disease progression, including inflammation, fibrosis, and eventually cirrhosis, cancer, and liver failure [5,8–12].

In normal individuals, up to 95% of BAs returns to the liver through enterohepatic circulation, with only a small portion of the overall pool derived from newly synthesized BAs [13]. In contrast, BA synthesis seems to play a significant role in hepatic injury under cholestatic conditions, as suppression of hepatic BA synthesis following administration of an engineered form of FGF19 led to dramatic protection of the liver from cholestasis [14]. FGF19 mRNA expression is absent in normal liver and BA synthesis is regulated by FGF19 secreted from the ileum [15–17,18]. On the other hand, FGF19 mRNA expression in the liver was reported to be increased by both extrahepatic and intrahepatic cholestasis [17,19]. The impact of this upregulated hepatic FGF19 expression on BA synthesis is still unclear and the overall role of circulating FGF19 and BA synthesis in cholestatic state needs to be further investigated. These findings may have implications for the potential therapeutic benefits derived from pharmacological inhibition of BA synthesis by modulating the expression and/or activity of CYP7A1. In this report, BA synthesis was directly quantified by measuring serum concentrations of 7alpha-hydroxycholest-4-en-3-one (C4), along with serum FGF19 and other relevant parameters, in patients with PBC and PBC with cirrhosis. The analysis of the relationships between circulating FGF19, BA synthesis, cholestasis and prognosis presented in this report further an understanding of PBC pathophysiology and support the therapeutic potential of blocking hepatic BA synthesis.

## Patients and methods

### Patients

Diagnosis and follow up of all patients included in the study were conducted at the third affiliated hospital of Sun Yat-Sen University between 2014 and 2015. Hospital Ethics Committee of the Third Affiliated Hospital of Sun Yat-sen University specifically approved the current study. PBC was diagnosed according to the AASLD Practice Guideline [1]. The diagnosis of cirrhosis was based on either liver biopsy or an imaging examination such as computed tomography, magnetic resonance imaging or ultrasonography. Patients were treated for PBC by daily administration of UDCA treatment at a daily dose of 13 to 15 mg/kg. Ten subjects with no clinical findings, as reflected by medical history, physical examination and serum biochemistry, were included in the study as healthy controls. Blood samples were obtained with the approval of hospital ethics committee and written consent from patients. Biochemical parameters were measured by standard clinical laboratory methods. Response to UDCA treatment was defined according to the “Paris Criteria” [20] and prognosis of the patients is represented by their Mayo Risk Score (MRC), calculated according to Dickson ER et al [21].

### Measurement of serum FGF19 and C4

Serum FGF19 levels were measured using a sandwich enzyme-linked immunosorbent assay (ELISA) (Biovendor, USA), according to the manufacturer’s instructions. C4 concentrations were measured by The Immunochemical Core Laboratory at Mayo Clinic in Rochester, MN according to methods published previously [22]. The method has an analytic measurement range from 0.5 ng/mL to 200 ng/mL.

### Statistical analysis

The baseline demographic and clinical characteristics are presented as percentage, or mean values with standard error of the mean (SEM); statistical comparisons between data sets were performed using χ^2^ or Fisher’s exact tests for categorical data and Student’s t or Mann—Whitney U tests for continuous data, as appropriate. Comparisons of biochemical values before and after 1 year of UDCA treatment were made using the Wilcoxon signed-rank test. The Spearman rank or Pearson’s correlation method was used for correlation analysis of data sets. To identify independent relationships and adjust the effects of covariates, multiple linear regression analyses were performed, including evaluation of all parameters showing highly significant correlations in the univariate analysis as covariates. The statistical analyses were calculated using SPSS statistics version 19 (IBM, Armonk, NY, USA). All analyses were two-sided and P-values <0.05 were considered statistically significant.

## Results

### Clinical and laboratory characteristics of patients with PBC and healthy control

Forty-four PBC patients (27 non-cirrhotic and 17 cirrhotic) and 10 healthy subjects were enrolled in the study (Table 1). As compared to healthy control subjects, serum levels of the liver enzymes (ALP, ALT, AST and GGT), bilirubin (TBIL and DBIL), total bile acids (TBA), IgG and IgM were significantly elevated in PBC patients, independent of cirrhosis. In contrast, serum levels of albumin (ALB), globulin (GLB), hemoglobin (Hb) and platelets (PLT) were significantly reduced in PBC patients, relative to healthy volunteers. In addition, concentrations of C-reactive protein (CRP) and prothrombin time (PT) were significantly increased in cirrhotic PBC patients, as compared to healthy controls. Furthermore, levels of TBA, PT and Mayo Risk Score were found to be significantly higher in cirrhotic PBC patients as compared to non-cirrhotic PBC patients.

**Table 1:** Clinical and laboratory characteristics of patients with PBC and healthy controls.

Feature	Healthy control (N = 10)	Non-cirrhotic PBC (N = 27)	Cirrhotic PBC (N = 27)
**Age (years)**	53 ± 5.1	52.0 ± 2.0	55.6 ± 2.7
**Gender (M/F)**	6/4	3/24	6/11
**ALP (U/L)**	54.9 ± 4.7	305.5 ± 39.7*	280.9 ± 29.5*
**ALT (U/L)**	16.3 ± 1.2	110.9 ± 16.8*	73.9 ± 14.2*
**AST (U/L)**	22.2 ± 1.2	114.0 ±15.1*	94.5 ± 12.4*
**GGT (U/L)**	26.9 ± 5.2	484.9 ± 84.2*	330.1 ± 84.0*
**TBIL (umol/l)**	12.3 ± 1.6	58.2 ± 12.1*	86.2 ± 19.1*
**DBIL (umol/l)**	3.9 ± 0.5	42.3 ± 10.5*	61.7 ± 14.9*
**TBA (umol/l)**	2.6 ± 0.5	80.3 ± 16.2*	134.8 ± 22.2*^#^
**CRP (mg/L)**	3.4 ± 0.5	8.4 ± 2.1	16.4 ± 4.7*
**ALB (g/L)**	44.4 ± 0.7	37.1 ± 1.0*	34.5 ± 1.3*
**GLB (g/L)**	27.1 ± 1.2	34.8 ± 1.2*	37.0 ± 2.4*
**PT (sec)**	13.0 ± 0.2	13.4 ± 1.0	15.6 ± 1.1*^#^
**Hb (g/L)**	139.5 ± 2.4	116.4 ± 3.9*	105.5 ± 6.2*
**PLT (10e9/L)**	234.4 ± 15.2	184.7 ± 15.3	137.7 ± 23.7*
**IgG (g/L)**	12.4 ± 0.8	17.9 ± 1.2*	21.6 ± 2.2*
**IgM (g/L)**	1.2 ± 0.1	3.7 ± 0.7*	2.5 ± 0.3*
**Mayo Risk Score**	-	5.5 ± 0.3	6.6 ± 0.4^#^

*P < 0.05 when compared to healthy control (Student t-test)

^#^P < 0.05 when compared to no-cirrhotic PBC (Student t-test)

### Serum FGF19 and C4 concentrations

**Fig 1:**
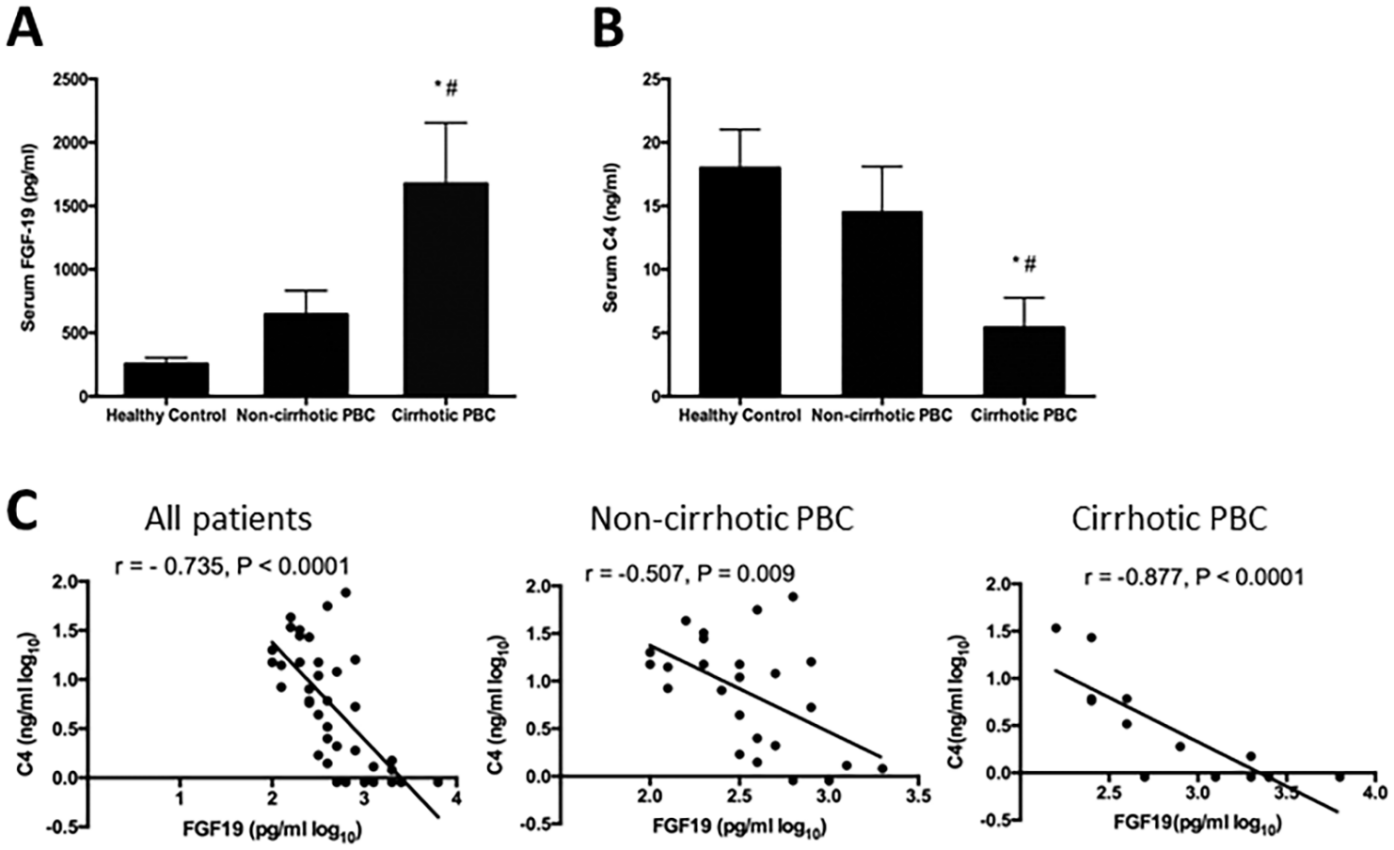
Serum FGF19 and C4 concentrations. (A) Serum FGF19 concentrations in patients with cirrhotic PBC were significantly higher as compared to healthy control (p < 0.05) and to patients with non-cirrhotic PBC (p < 0.05); (B) Serum C4 concentrations in patients with cirrhotic PBC were significantly lower as compared to healthy control (p < 0.05) and to patients with non-cirrhotic PBC (p < 0.05); (C) Strong negative correlation between serum FGF19 and C4 in all PBC patients (r = -0.735, p < 0.0001), non-cirrhotic PBC patients (r = -0.507, P < 0.009) and cirrhotic PBC patients (r = -0.877, P < 0.0001).

Serum FGF19 concentrations in patients with non-cirrhotic PBC were numerically higher, but not statistically different, from levels measured in healthy control subjects (645.6 ± 187.9 vs. 255.9 ± 48.5 pg/ml; p > 0.05) (Fig 1A). In contrast, serum FGF19 concentrations in patients with cirrhotic PBC were significantly higher as compared to either healthy volunteer subjects (1674.0 ± 480.1 vs. 255.9 ± 48.5 ng/ml; p < 0.05) or patients diagnosed with non-cirrhotic PBC (1674.0 ± 480.1 vs. 645.6 ± 187.9 ng/ml; p < 0.05) (Fig 1A).

Serum C4 concentrations in patients with non-cirrhotic PBC were not different from that of healthy control (15.7 ± 3.8 vs. 18.0 ± 3.0 ng/ml; p > 0.05) (Fig 1B). However, serum C4 concentrations in patients with cirrhotic PBC were significantly lower compared to healthy control subjects (6.1 ± 2.6 vs. 18.0 ± 3.0 ng/ml; p < 0.05) and to patients with non-cirrhotic PBC (6.1 ± 2.6 vs. 15.7 ± 3.8 ng/ml; p < 0.05) (Fig 1B).

Based on these data, we observe a strong negative correlation (r = -0.735, p < 0.0001) between serum levels of FGF19 and C4 in all PBC patients (with or without cirrhosis) (Fig 1C). Interestingly, this correlation is much stronger (r = -0.877, P < 0.0001) in cirrhotic PBC patients as compared to that observed in non-cirrhotic PBC patients (r = -0.507, P < 0.009).

### Serum FGF19 and C4 concentrations correlate with cholestasis and inflammatory response

**Fig 2:**
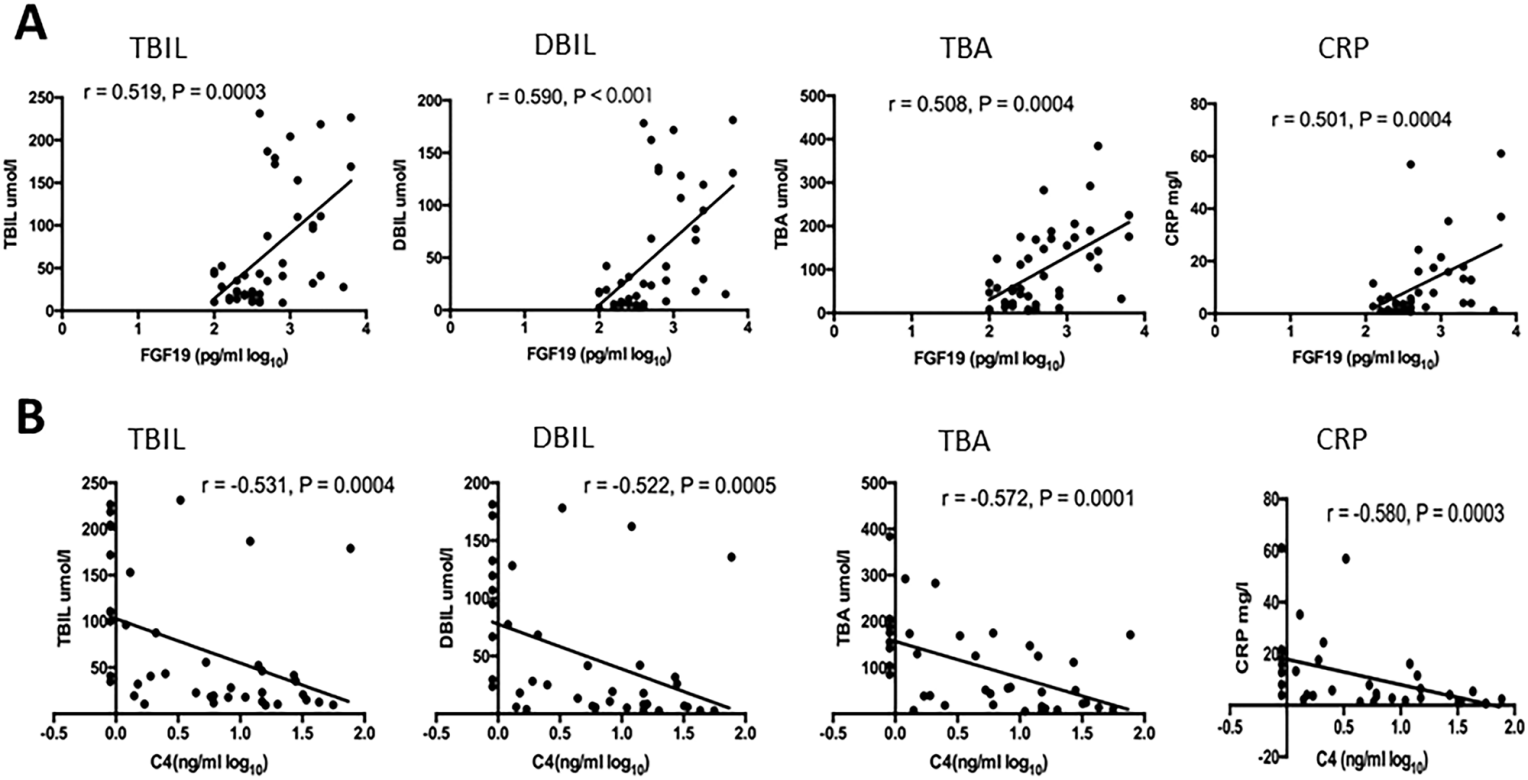
Correlation of serum FGF19 and C4 concentrations with cholestasis and inflammatory response. (A) Serum FGF19 was positively correlated with TBIL (r = 0.519, p = 0.003), DBIL (r = 0.590, p < 0.001), TBA (r = 0.508, p = 0.0004), and CRP (r = 0.501, p = 0.0004); (B) Serum C4 was negatively correlated with TBIL (r = 0.531, p = 0.0004), DBIL (r = 0.522, p = 0.0005), TBA (r = 0.572, p = 0.0001), and CRP (r = -0.580, p = 0.0003).

Serum levels of TBIL, DBIL and TBA are typically used as indicators of cholestasis in patients. Notably, we observe positive correlations between concentrations of TBIL and FGF19 (r = 0.519, p = 0.003), DBIL and FGF19 (r = 0.590, p < 0.001), and TBA and FGF19 (r = 0.508, p = 0.0004) (Fig 2A) in the serum of PBC patients participating in this study. On the other hand, serum levels of TBIL and C4 (r = 0.531, p = 0.0004), DBIL and C4 (r = 0.522, p = 0.0005), and TBA and C4 (r = 0.572, p = 0.0001) are negatively correlated in these patients (Fig 2B). Multivariate analysis of these data reveals that both TBIL and DBIL are independent variables (P = 0.002 and P = 0.003) of serum FGF-19 when FGF-19 was independent variable (P = 0.043) of serum C4 levels in PBC patients.

Serum levels of CRP, an acute-phase protein expressed in the liver, rise in response to inflammation and is used as an indicator of inflammatory response. As shown in Fig 2, we observe a positive correlation between concentrations of CRP and FGF19 (r = 0.501, p = 0.0004) (Fig 2A) and a negative correlation between CRP and C4 levels (r = -0.580, p = 0.0003)(Fig 2B) in PBC patients.

### Serum FGF19 and C4 concentrations, liver enzymes, patient prognosis and UDCA responsiveness

**Fig 3:**
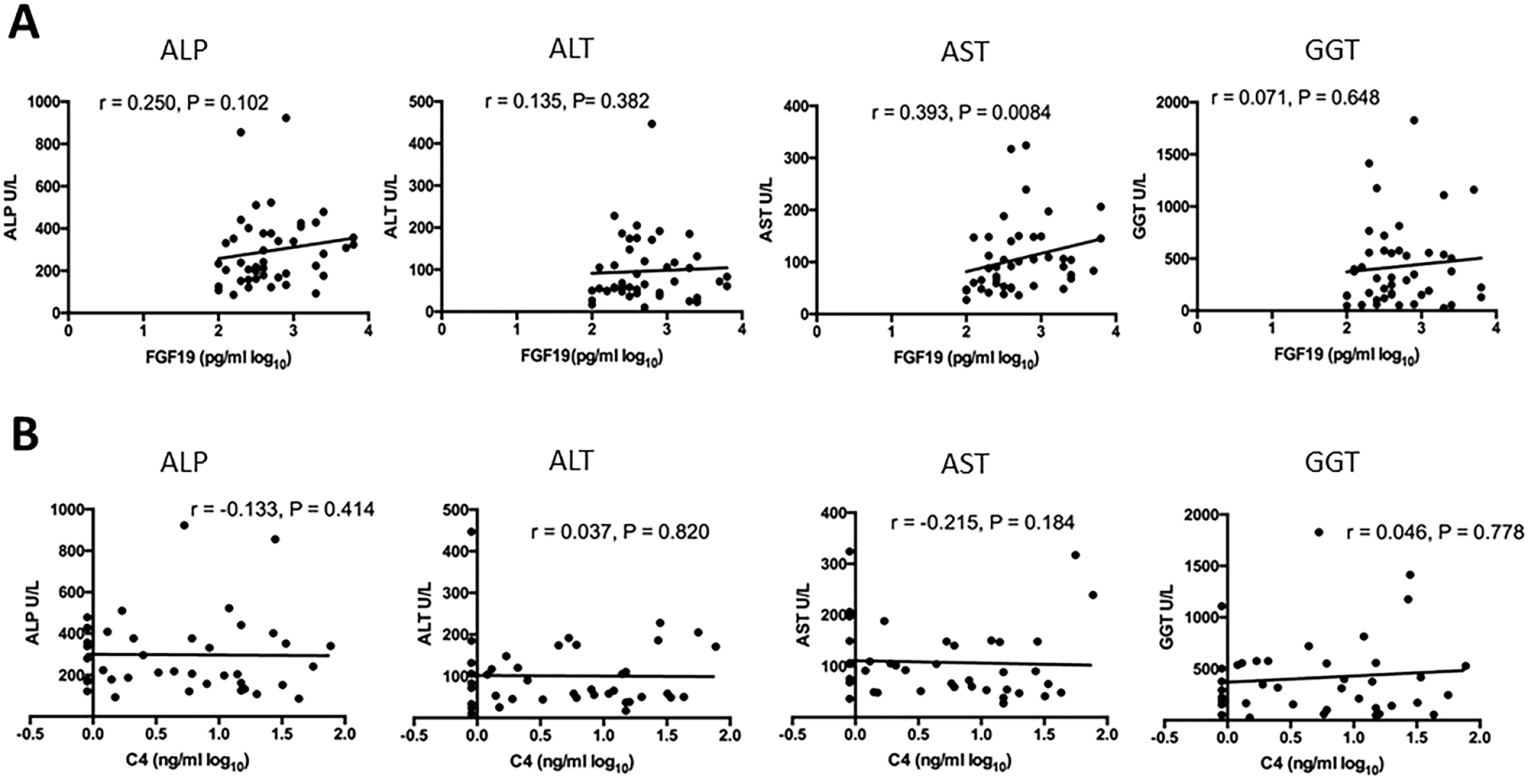
Correlation of serum FGF19 and C4 concentrations with liver enzymes. (A) Correlation of serum FGF19 with ALP, ALT, AST or GGT; (B) Correlation of serum C4 with ALP, ALT, AST or GGT.

Other than a moderately positive correlation between AST and serum FGF19 (r = 0.393, p = 0.0084) (Fig 3A), no correlations between other liver enzymes and serum FGF19 were detected in the PBC patients. Similarly, no correlations between any of the liver enzymes and serum C4 (Fig 3B) could be demonstrated in the cohorts of PBC patients examined in this study.

**Fig 4:**
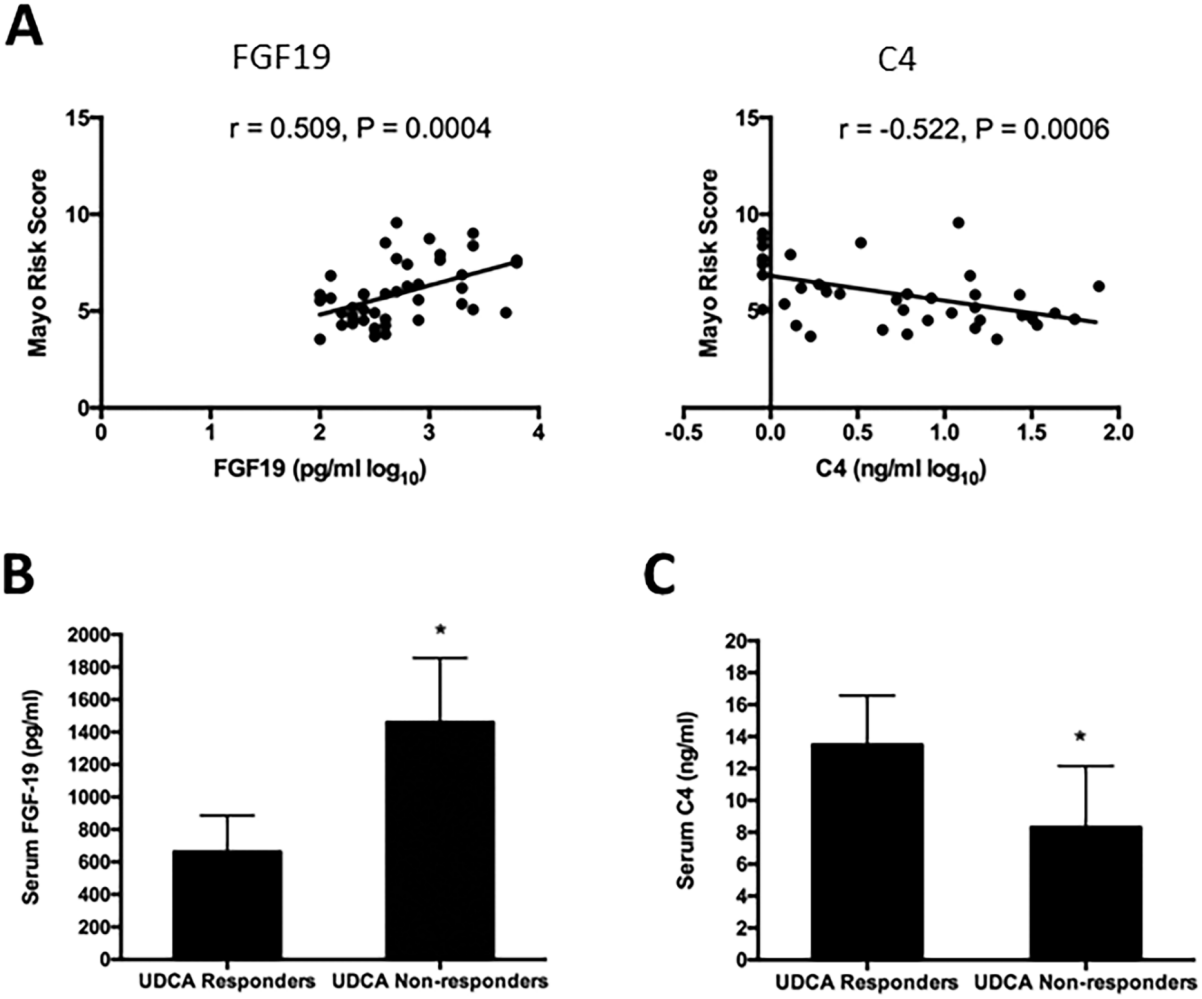
Correlation of serum FGF19 and C4 concentrations with Mayo Risk Score and serum FGF19 and C4 concentrations in UDCA responders and non-responders. (A) Serum FGF19 was positively correlated with Mayo risk score (r = 0.509, p = 0.0004) while serum C4 was negatively correlated with Mayo Risk Score (r = -0.522, p = 0.0006); (B) Serum FGF-19 concentrations in UDCA responders were significantly higher than that in UDCA non-responders (p = 0.018); (C) Serum C4 concentrations in UDCA responders were significantly lower than that in UDCA non-responders (p = 0.021).

Based on a calculation involving a collection of potential risk factors, including patient’s age, serum bilirubin and albumin concentrations, prothrombin time, and presence of peripheral edema and antidiuretic therapy, the Mayo Risk Score has been commonly used to evaluate the prognosis of PBC patients [19]. In this study, we observe a clear positive correlation between the Mayo Risk Score and serum FGF19 levels (r = 0.509, p = 0.0004)(Fig 4A) and a negative correlations between Mayo Risk Score and serum C4 (r = -0.522, p = 0.0006)(Fig 4A) in PBC patients.

Among the 44 PBC patients, there were 23 UDCA responders and 21 UDCA non-responders. Notably, serum FGF-19 concentrations were significantly higher (p = 0.018) in PBC patients that failed to respond to UDCA treatment (1459.26±397.16), compared to the UDCA responders (662.74±223.56) (Fig 4B). Conversely, serum C4 concentrations were significantly lower in UDCA non-responders, as compared to the levels found in UDCA responders (8.30±3.86 vs. 13.47±3.10, p = 0.021) (Fig 4C). Clinical and laboratory characteristics of PBC patients are shown in Table 2 as a function of their response to UDCA.

**Table 2:** Clinical and laboratory characteristics of UDCA responders and non- responders.

Feature	UDCA responders (N = 23)	UDCA non-responders (N = 21)	P value
**Age (years**	54.8 ± 2.3	52.0 ± 2.0	0.352
**Gender (M/F)**	19/4	3/24	0.716
**Cirrhosis**	5	12	0.016
**FGF19 (pg/ml)**	662.7 ± 223.6	1459.3 ± 397.2	0.018
**C4 (ng/ml)**	13.5 ± 3.1	8.3 ± 3.9	0.021
**ALT (UL**	94.1 ± 19.2	99.3 ± 3.9	0.488
**AST (U/L)**	96.0 ± 16.4	117.9 ± 12.3	0.041
**TBIL (umol/l)**	33.0 ± 7.6	108.4 ± 16.9	0.000
**DBIL (umol/l)**	19.7 ± 6.3	82.7 ± 13.6	0.000
**GGT (U/L)**	370.0 ± 87.1	485.3 ± 83.8	0.160
**ALP (U/L)**	239.0 ± 36.7	358.5 ± 34.8	0.001
**TBA (umol/l)**	59.0 ± 15.4	147.6 ± 18.5	0.000
**ALB (g/L)**	39.3 ± 0.9	32.6 ± 0.9	0.000
**GLB (g/L)**	35.2 ± 1.5	36.1 ± 1.9	0.719
**PT (sec)**	13.0 ± 0.3	15.7 ± 1.1	0.034
**Hb (g/L)**	126.4 ± 3.6	96.6± 4.0	0.000
**PLT (10e9/L)**	171.6 ± 18.7	161.0 ± 19.6	0.740
**IgG (g/L)**	18.1 ± 1.2	20.2 ± 1.8	0.718
**IgM (g/L)**	3.4 ± 0.6	3.2 ± 0.7	0.945
**CRP (mg/L)**	4.4 ± 0.9	18.7 ± 4.0	0.001
**Mayo Risk Score**	5.1 ± 0.2	6.8 ± 0.4	0.000

## Discussion

As part of a complex enterohepatic feedback mechanism, circulating FGF19 regulates BA synthesis in the liver by suppressing expression of the gene that encodes CYP7A1, the rate-limiting enzyme in bile acid synthesis [16]. An earlier report failed to determine whether there is any significant difference in serum FGF19 concentrations between PBC patients and their healthy counterparts [19]. For the first time, the current report shows that the observed FGF19 concentrations in non-cirrhotic PBC patients were numerically higher than that of healthy individuals, and the concentrations in cirrhotic PBC patients were substantially higher as compared to either healthy control or non-cirrhotic PBC patients (Fig 1A). Corresponding to these changes, serum C4 concentrations in non-cirrhotic PBC patients were numerically lower than that of healthy subjects (Fig 1B) and the concentrations in cirrhotic PBC patients were substantially lower as compared to either healthy control or non-cirrhotic PBC patients (Fig 1B).

C4 is a direct intermediary product of CYP7A1 in the process of BA synthesis [23–24]. The temporal relationship between serum levels of C4 and FGF19 under physiological conditions supports the notion that circulating FGF19 regulates BA synthesis through CYP7A1 [20]. On the other hand, the role of FGF19 in the regulation of BA synthesis is less well established in states of chronic cholestasis. Schaap FG *et al*. demonstrated high levels of FGF19 expression in the liver of patients with extrahepatic cholestasis [17], and more recently, Wunsch E *et al*. showed that hepatic FGF19 expression was significantly increased in both non-cirrhotic (9-folds of normal) and cirrhotic (69-folds of normal) PBC patients [19]. Wunsch E *et al*., suggest that CYP7A1 regulation involves two parallel pathways, both of which operate independently of enterocyte-derived FGF19. In particular, one of these regulatory circuits is governed by the hepatic FXR/SHP axis, whereas the second acts via a not yet defined, paracrine/autocrine action of hepatic FGF19 [19]. However, this model seems to be in conflict with our findings. The strong correlation between serum levels of FGF19 and C4 in the cholestatic state suggests the regulation of BA synthesis (mediated through CYP7A1) is still dominated by circulating FGF19. The strong correlations between serum FGF19 and C4 and the degree of cholestasis, as indicated by bilirubin and TBA (Fig 2), further support this notion. Taken together, these data support that the physiological FGF19 feedback mechanism of BA synthesis regulation remains intact in a state of chronic cholestasis. It is possible that hepatic FGF19 over-expression contributes to the overall pool of circulating FGF19, and it is this pool that is responsible for modulating BA synthesis in the liver. As demonstrated both by CDCA-mediated induction of ileal FGF19 expression [18] and pharmacological administration of M70, a non-tumerigenic FGF19 analog in humans [14], circulating FGF19 efficiently suppresses CYP7A1 expression and acts as a powerful regulator of BA synthesis. The mechanisms by which FGF19 regulates BA synthesis and composition are complex and dynamic and pathophysiological effects of chronic cholestasis add further complexity. In addition to enterocytes and hepatocytes, FGF19 can also be derived from the cholangiocytes of the intrahepatic biliary tree [25]. Clearly, additional investigation regarding the various sources of elevated levels of circulating FGF19 in PBC patients is warranted.

As indicated by serum C4 concentrations, BA synthesis was clearly suppressed in cirrhotic PBC patients (Fig 1A). Targeting these patients for further suppression of BA synthesis by pharmacological intervention, *e*.*g*., the engineered FGF19 derivative M70, may not represent an effective approach to treatment. On the other hand, the majority of PBC patients have normal, or at least, significant levels of C4 and BA synthesis, and given the central role of the accumulated hepatic BAs in the pathogenesis of PBC [5–7,14], suppressing BA synthesis may provide significant therapeutic benefits. Indeed, NGM282, an engineered form of human FGF19, has been shown to robustly decrease serum C4 levels and significantly reduce ALP levels in a recent phase II clinical study of PBC patients unresponsive to UDCA treatment [26].

It is clear that there is a strong correlation between BA synthesis and the severity of cholestasis as well as BA synthesis and inflammatory response (Fig 2B) in PBC patients. In contrast, with the exception of the modest negative correlation between serum FGF19 and AST, neither rates of BA synthesis (as indicated by C4 levels) nor serum FGF19 levels showed any correlation with any of the liver enzymes (Fig 3). These observations seem to be consistent with previous findings [15]. One potential explanation is that the levels of liver enzymes (or the degree of liver injury) depend mainly on the accumulation of hepatic BAs, and as the degree of cholestasis and serum levels of bilirubin and TBA are cross sectional measurements, they may not accurately reflect the degree of hepatic BA accumulation in PBC patients.

Consistent with previous findings in PBC patients [19], we observe a strong positive correlation between serum FGF19 and the Mayo Risk Score and a negative correlation between serum C4 and Mayo Risk Score (Fig 4A). In addition, analysis of the UDCA response showed that UDCA non-responders had significantly higher levels of serum FGF19 and lower levels of C4 (Fig 4B and 4C). In both situations, the increase of FGF19 is likely an indirect reflection of the cholestatic state of the patients. Collectively, these findings support that serum concentrations of FGF19 and C4 seem to reflect the disease state and could serve as potential prognostic indicators or markers in PBC. Research in treatment-naïve PBC patients may provide a deeper understanding of the relationships among serum markers of bile acid synthesis, cholestasis and disease state.

In conclusion, our findings demonstrate that circulating levels of FGF19 are proportionally increased in response to the degree of cholestasis in patients diagnosed with PBC. These increased levels of circulating FGF19 lead to a suppression of BA synthesis. In addition, measurement of circulating levels of FGF19 or C4, along with Mayo Risk Score, could potentially be used to determine the prognosis of PBC patients. Although FGF19-mediated suppression of BA synthesis appears to operate in these patients, this feedback mechanism seems to be insufficiently robust to counter or reverse disease progression. Activating this mechanism by augmenting resident FGF19 through pharmacological intervention with FGF19 or a suitable mimetic, could provide a powerful and novel therapeutic approach to treat patients with PBC.
